# Elderberry Supplementation Reduces Cold Duration and Symptoms in Air-Travellers: A Randomized, Double-Blind Placebo-Controlled Clinical Trial

**DOI:** 10.3390/nu8040182

**Published:** 2016-03-24

**Authors:** Evelin Tiralongo, Shirley S. Wee, Rodney A. Lea

**Affiliations:** 1 School of Pharmacy, Griffith University, Gold Coast campus, Queensland 4222, Australia; 2 Menzies Health Institute Queensland, Griffith University, Gold Coast campus, Queensland 4222, Australia; s.wee@griffith.edu.au; 3 School of Allied Health Sciences, Griffith University, Gold Coast campus, Queensland 4222, Australia; 4 Genomics Research Centre, Institute of Health and Biomedical Innovation, Queensland University of Technology, Queensland 4000, Australia; rodney.a.lea@gmail.com

**Keywords:** elderberry, travel, cold symptoms, clinical trial, nutritional supplements, complementary medicines, sambucus, physical health

## Abstract

Intercontinental air travel can be stressful, especially for respiratory health. Elderberries have been used traditionally, and in some observational and clinical studies, as supportive agents against the common cold and influenza. This randomized, double-blind placebo-controlled clinical trial of 312 economy class passengers travelling from Australia to an overseas destination aimed to investigate if a standardised membrane filtered elderberry (*Sambucus nigra* L.) extract has beneficial effects on physical, especially respiratory, and mental health. Cold episodes, cold duration and symptoms were noted in a daily diary and assessed using the Jackson score. Participants also completed three surveys containing questions regarding upper respiratory symptoms (WURSS-21) and quality of life (SF-12) at baseline, just before travel and at 4-days after travel. Most cold episodes occurred in the placebo group (17 *vs*. 12), however the difference was not significant (*p* = 0.4). Placebo group participants had a significantly longer duration of cold episode days (117 *vs*. 57, *p* = 0.02) and the average symptom score over these days was also significantly higher (583 *vs*. 247, *p* = 0.05). These data suggest a significant reduction of cold duration and severity in air travelers. More research is warranted to confirm this effect and to evaluate elderberry’s physical and mental health benefits.

## 1. Introduction

Air travel, especially on intercontinental flights can be stressful, thus putting extra strain on passenger’s physical and psychological health [1]. Studies have shown that passengers’ well-being is influenced by the cabin environment such as cabin ozone concentration and oxygen pressure, motion or vibration and oil additives used in aircraft engines [2,3]. Fatigue, impairment of immunity as well as increased stress and mental changes has been reported during and after long-distance flights [4,5,6]. A recent review of medical in-flight events lists respiratory symptoms among the most common medical complaints reported [7]. Not surprisingly, many studies have investigated the occurrence of nasal dryness and the increased risk of developing upper respiratory disorders such as allergic rhinitis and attracting virus or bacteria induced respiratory infections during long-haul air travel [8,9]. Moreover, an added risk of spreading respiratory diseases, including influenza, aboard commercial flights exists [10].

Recently, for the first time a herbal medicine, echinacea, was trialled in travellers and showed protective effect against the development of respiratory symptoms during travel involving long-haul flights [11]. Following travellers return from overseas, participants using echinacea displayed a lower respiratory symptom score and the overall percentage of participants affected by respiratory disease symptoms was marginally lower in the Echinacea group compared to placebo.

However, there are other herbs that have traditionally been used to treat respiratory symptoms and aid in the recuperation from a cold. Black elderberries for example, are well known to be supportive agents against common cold and flu like symptoms and have been used for centuries [12]. Interestingly, a non-travel related clinical trial just revealed that a combination of echinacea herb and root extract supplemented with elderberry (*Sambucus nigra* L.) can be as effective as the conventional antiviral medicine oseltamivir for the early treatment of influenza [13].

Elderberries have shown antibacterial [14] and antiviral activities in *in vitro* [15]. Two clinical trials using a liquid elderberry extract (Sambucol^®^, Israel) showed a reduction in symptoms and duration of influenza infection [16]. A pilot trial with elderberry extract lozenges (HerbalScience, Singapore) also confirmed a beneficial effect on severity and duration of cold and flu like symptoms [17].

In recent times, elderberry has gained popularity in research and the wider community due to its reported antioxidant [18], antidiabetic [19], anti-inflammatory and immune-modulating [20], as well as antidepressant [21] properties. The berries are dark violet-black drupes which grow in clusters and owe their colour to the anthocyanins; a group of phenolic compounds which, amongst flavonoids, are abundant in elderberries and considered the active constituents of the fruits [22]. However, elderberries also contain a variety of nutrients ranging from various vitamins (A, B_1_, B_2_, B_6_, B_9_, C and E), trace elements such as Cu, Zn, Fe and minerals such as K, Ca and Mg to phytochemicals such as carotenoids, phytosterols and polyphenols. These additional constituents and activities make elderberries a likely candidate for beneficial nutritional and/or medical supplementation not only for respiratory, but also for cardiovascular and mental health, all of which may be affected during travel.

Given that elderberry supplementation has never been investigated so far for its possible beneficial effects in air-travelers, we conducted a randomized controlled trial aimed at identifying whether capsules containing a proprietary membrane filtered Elderberry extract (Iprona, Italy), standardized to polyphenols, are effective in preventing respiratory symptoms, but also if they positively impact on the overall physical and mental health of travelers when using long-haul, commercial flights as means of transport.

## 2. Experimental Section

### 2.1. Study Participants and Randomisation

Participants were recruited through travel agencies; radio, newspaper and TV advertisements, and emails circulated to all staff and students at a university on the Gold Coast, Australia. All participants gave written informed consent before participating in the study. Volunteers were included if they were a minimum of 18 years of age and in good general health. Volunteers were excluded if they were currently in another clinical trial or were in one less than 30 days ago, had a known plant allergy, were suffering from respiratory diseases (e.g., asthma, chronic obstructive pulmonary disease), had any other condition that could compromise the study or the participants health (e.g., autoimmune disease, cystic fibrosis), had received flu vaccination less than 10 days of starting the trial, were lactating, pregnant or planning to become pregnant or were on regular treatment with antibiotics, corticosteroids, antihistamines, antivirals, non-steroidal anti-inflammatory drugs, anticancer drugs and immune-suppressants.

Three hundred and twenty five volunteers met the inclusion criteria and were randomly assigned to trial capsules. The random allocation sequence provided by an independent consultant was computer generated using a randomisation plan from www.randomization.com with randomisation in blocks of 10. A list of consecutive study numbers was generated. Treatment groups were allocated by trial staff, but the allocation was concealed by assigning each participant with a unique number. Participants, chief investigators and trial staff were blinded to group allocation.

### 2.2. Study Design

A randomised, double-blind placebo controlled clinical trial was conducted between April 2013 and December 2014 in Australia with economy class passengers travelling to an overseas destination, on a minimum of 7 h flight, *i.e.*, Australia-Asia, less than a 12-h stopover and a minimum of four days stay at the destination.

The clinical trial received ethical approval from the institutional Human Research Ethics Committee (PHM/08/12/HREC, January 2013) and was registered with the Australian New Zealand Clinical Trials Registry http://www.anzctr.org.au (ACTRN12612001301853).

**Figure 1:**
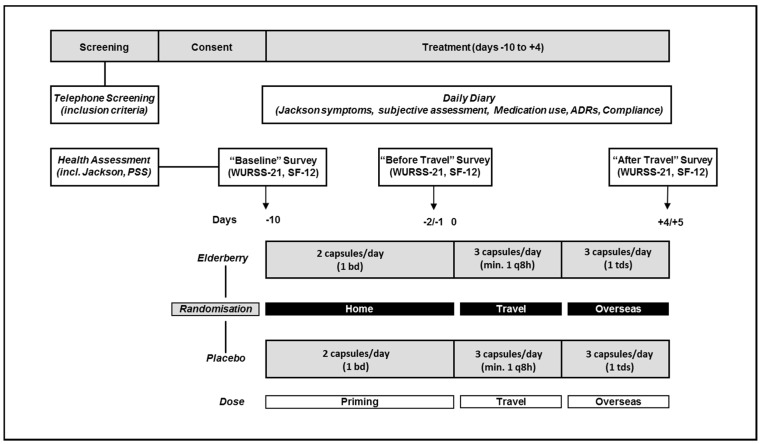
Study design.

Figure 1 outlines the study design. For all participants treatment would commence 10 days before flying overseas and would be completed five days after arriving at the travel destination. The actual treatment time varied between participants depending on their actual travel duration ranging from 15 to 16 days.

Participants completed surveys at baseline (−10 days), before travel (−2 days) and after travel (+4/5 days), as well as recording cold symptoms in a daily diary. The health assessment included the Perceived Stress Scale (PSS). Surveys included assessments of general well-being (SF-12 acute) and quality of life related to respiratory symptoms (Wisconsin Upper Respiratory Symptom Survey, WURSS-21) (Figure 1). Cold diagnosis was assessed by measuring the Jackson Score.

### 2.3. Elderberry Supplementation

The elderberry extract used in this trial is a proprietary membrane filtered elderberry (*Sambucus nigra* L., Haschberg variety, Steiermark region in Austria) extract produced by Iprona AG, Lana (BZ), Italy, under their BerryPharma^®^ brand, and has been shown to possess antimicrobial activity against human respiratory bacterial pathogens (Gram-positive bacteria of *Streptococcus pyogenes* and group C and G Streptococci, and the Gram-negative bacterium *Branhamella catarrhalis*), and also displays an inhibitory effect on the propagation of human pathogenic influenza viruses [14].

This extract is currently used in the production of various products marketed in Asia, the US and Europe. The elderberry capsules used in this trial were produced by Plantafood in Germany in accordance with the principles and guidelines of Good Manufacturing Practice. They contain: 300 mg of elderberry extract (22% polyphenols (*i.e.*, quercetin and its glycosides, rutin), 15% anthocyanins (*i.e.*, cyanidin and pelargonidin glycosides) and 150 mg of rice flour. The 300 mg elderberry extract also contain several mineral, trace elements and vitamins including relatively high levels of magnesium 1.19 mg (Mg: 3.97 mg/g).

Placebo capsules were manufactured to match the elderberry capsules in size, excipients and appearance. Capsules were supplied in identical blister packs with identical labelling. Labelling only identified the participant number.

Travellers took elderberry capsules or placebo from 10 days before travel (2 capsules/day, priming dose (−10 to −2 days) and from 1 day before leaving home until 4/5 days after arriving at the destination (3 capsules/day, overseas dose (−1 to +5 days) (Figure 1). The dose selection was based on dosages used by popular elderberry products which range from 650 mg to 1500 mg per day). The trial dose used here is similar with participants required to take two capsules per day (600 mg) during the priming phase (before travel, −10 until −2 days) and three capsules per day (900 mg) while travelling and overseas (−1 until +4 days).

Compliance was assessed by calculating the percentage of capsules taken against total capsules expected to be taken during the treatment period.

### 2.4. Clinical Outcome Measurements and Statistical Analysis

Jackson Score: Over the entire study period, all participants maintained a diary. This was to record cold symptoms, as well as additional health issues or disease symptoms and additional medication taken. The diary helped participants with recalling information when completing the surveys and allowed researchers to identify possible inconsistencies in data documentation.

The main question the participant answered in the diary, “Do you believe you have a cold today?” was answered yes or no. During acute colds, the symptoms “headache”, “chilliness”, “sneezing”, “nasal obstruction”, “nasal discharge”, “sore throat”, “cough”, and “malaise” were rated on a 4-point Likert scale with 0 or no entry = absence, 1 = mild, 2 = moderate, and 3 = severe symptoms. In addition, the participant indicated in the diary the daily intake of concomitant medication and/or therapy. This matrix was based on the work by Jackson and colleagues, who described the clinical features and symptoms of a virally induced common cold [23]. Their definition is currently accepted as the most valid method for differentiating a cold from isolated symptoms (like hay fever or allergies) that do not develop into the clinical picture of a cold. Thus, a cold episode was defined as a minimal total symptom score of 14 (summed over a minimum of six consecutive days), and the participants believed they had a cold and/or reported rhinorrhoea that lasted for *≥* 3 days. A set of three predefined prophylactic variables were analyzed in a confirmatory manner: (i) the total number of cold episodes; (ii) cumulative episode days; and (iii) co-medicated cold episodes. The three parameters were analyzed individually with a Chi-square test or Fisher’s exact test to determine whether the proportions of cumulated events (*i.e.*, cold episodes) in the treatment groups deviated from expectation under the null hypothesis of equal proportions among groups.

WURSS-21: In this study the upper respiratory symptom-related Quality of life (QoL) was measured using the questions from the 21-item Wisconsin Upper Respiratory Symptom Survey (WURSS-21) [24]. The WURSS-21 is a responsive, reliable and valid instrument for evaluating QoL outcomes related to respiratory illness, measuring all significant health-related dimensions that are negatively affected by the common cold [24,25]. It includes 10 items assessing symptoms, nine items assessing functional impairments and one item each assessing global severity and global change over the last 24 h, all of which are based on 7-point Likert-type severity scales. A previous validation of the instrument showed that a cumulative score should be calculated by summing the severity scores of the first 20 items with high severity scores indicating high symptom load. The mean WURSS-21 score was calculated and analyzed and compared between groups using ANOVA.

In addition, the minimal important difference (MID) for the WURSS-21 was calculated. The MID is the term generally used to quantify the minimum amount of positive change that patients perceive, and would accept an associated treatment as being beneficial or worth taking—a clinically significant effect [26]. For the WURSS-21 score, a MID of 10.3 was determined [27]. Therefore, individuals that presented with a respiratory disorder symptom score of 10.3 and above (RDS+) were compared in both groups at baseline, return and follow-up. Difference in the proportion of RDS+ individuals between groups at follow-up was statistically compared using a Chi-squared test and Fisher’s exact test. The amount of missing data was different among variables but on average was less than 10%. We analyzed observed data only, *i.e.*, did not impute data or conduct missing at random analyses.

SF-21: The SF-21 was utilised to assess general Quality of Life (QoL). The SF-12 is a generic measure and has been developed to provide a shorter, yet valid alternative to the SF-36, which has been seen by many health researchers as too long to administer [28]. The SF-12 is weighted and summed to provide easily interpretable scales for physical and mental health. Physical and Mental Health Composite Scores (PCS and MCS) are computed using the scores of twelve questions and range from 0 to 100, where a zero score indicates the lowest level of health measured by the scales and 100 indicates the highest level of health. All SF-21 variables were statistically analyzed using ANOVA to compare means.

PSS-SCALE: The Perceived Stress Scale (PSS) is the most widely used psychological instrument for measuring the perception of stress, the scale is based on feelings and thoughts during the last month. It has been validated to correlate with cold episodes [29]. It will be used to identify a possible sub-group of heightened stress participants and help analysing a possible correlation between increased stress and the effects of elderberry for this sub-group as opposed to the overall participant group. Individuals scoring > 14 points were considered “stressed” and as such selected for sub-group analysis.

### 2.5. Sample Size Calculation

The primary outcome measure for this study is the total number of cold episode days measured for the previous six days. A cold episode is defined by a Jackson score ≥14 and either (i) the participant believed they had a cold or (ii) reported rhinorrhea. In the placebo group we assumed a prevalence of cold episode days of 35% (235 days) over the 6-day survey period. This estimate is based on similar previous studies conducted by Jawad *et al*., 2012 [30] and Tiralongo *et al*., 2011 [11]. We also expected a prophylactic effect size (δ) of 0.5 (or a 2-fold decrease), this is an estimate based on the findings of an echinacea trial by Tiralongo *et al*., 2011 [11]. With an anticipated 2-fold prophylactic effect (δ = 0.5) we expected to observe a statistically significant reduction in episode days from 35% to 18% in the treatment group. Considering these parameters we estimated that a sample size of 280 (*n* = 140 in each arm) entering the study (ITT) would be required to achieve at least 80% power (β ≥ 0.8) to detect a δ ≥ 0.5 as statistically significant (α ≤ 0.05).

## 3. Results

**Figure 2:**
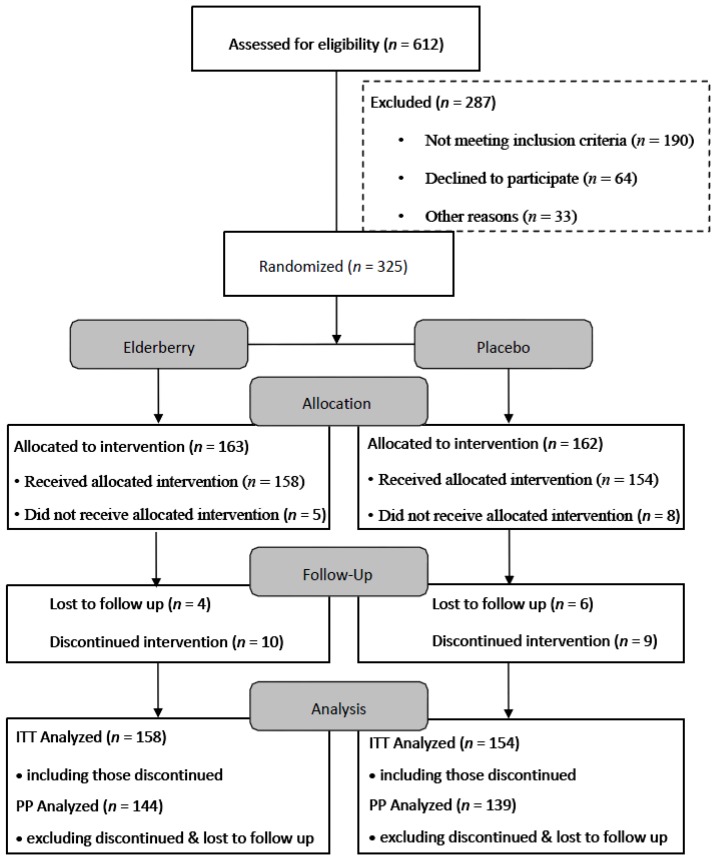
Flowchart of trial participants (ITT—Intention to treat analysis, PP—Per protocol analysis).

The flow of participants through the trial between April 2013 and December 2014 is summarised in Figure 2. Six hundred and twelve people were screened, with a number deemed ineligible by inclusion criteria *i.e.*, plant allergies, inappropriate destination and/or extended stopovers during travel (>12 h). Reasons for declining participation included not wanting to be on placebo, travel cancellation and personal circumstances. Of the 325 trial participants, 13 participants did not receive the trial medication due to various reasons (e.g., trial pack lost in mail, diagnosed with pneumonia, change of travel plans, change of mind and family emergency).

However, 312 completed the first survey. Thus Intention to treat (ITT) analysis was performed on 312 participants. Of the 312 participants analysed, 158 were assigned elderberry capsules and 154 were assigned placebo. The majority of the participants were women, non-smoker, on average 50 years old, travelled for holiday with a travel time of over 16 h (Table 1). The majority of participants indicated to be stressed (PSS > 14). About half of the participants received the flu vaccination. Participants in the placebo and active groups did not differ significantly at baseline. Thus, the two treatment groups can be considered reasonably well balanced at baseline.

To confirm that blinding was effective, all participants were asked to speculate whether they were taking elderberry or placebo. 80 participants (52%) identified themselves correctly in the placebo and 73 (46%) in the elderberry group. Thus, there was an even distribution of mismatches in the placebo and treatment group (*p* = 0.5) providing evidence of effective randomisation.

Analysis of the Jackson Score identified that 29 of 312 participants (~9%) suffered from a well-defined cold—17 of those were on placebo and 12 on elderberry. Although more cold episodes occurred in the placebo group, the difference between the groups was not significant (*p* = 0.2). The cold symptoms presented for approximately one third of participants (*n* = 11) before travel, for three during travel and for the majority of participants (*n* = 15) when they arrived overseas.

**Figure 3:**
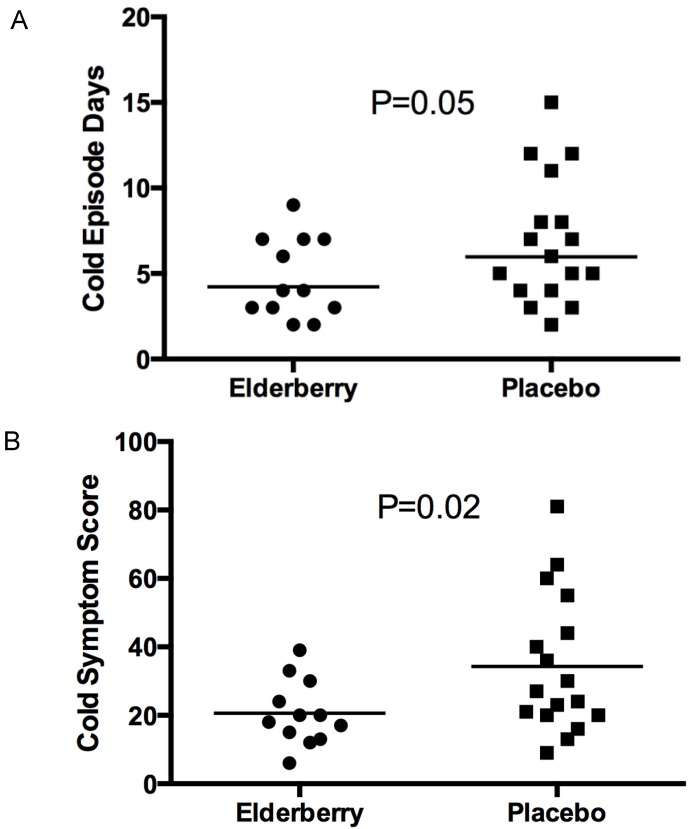
Cold episode days (**A**) and cold symptom score (**B**) of participants with a well-defined cold established from the Jackson Score.

However, the placebo group had a collective duration of 117 episode days; in comparison, the elderberry group had a significantly lower number of cold episode days (57, *p* = 0.05) (Figure 3A). Moreover, the symptom score in the placebo group over these days was 583, whereas in the elderberry group it was 247 (Figure 3B). The difference in symptom score between both groups was also significant (*p* = 0.02).

This indicates that a participant suffering from a cold episode while taking elderberry would on average experience a 2-day shorter duration of the cold (4.75 days *vs*. 6.88 days), and would experience a lower symptom severity (21 *vs*. 34) in comparison to participants suffering from a cold and taking placebo.

In both groups half the participants with a defined cold used co-medication to relieve symptoms. On average participants co-medicated for 1.5 days; the difference was not significant between groups (*p* = 0.9). The participants suffering from a cold took in total 25 conventional medicines, belonging to 10 different categories, and one complementary medicine. Some took one medicine, others up to four. The medicines used were: one nasal spray, four cold and flu tablets, seven analgesics, one antibiotic, three decongestants, one inhaler, one gargle/mouthwash, five lozenges, one cough syrup and one antihistamine. The complementary medicine used was an Indonesian product called Tolak angin.

The analysis of the WURSS data showed an increase of the mean WURSS-21 scores for elderberry and placebo participants from baseline to the end of the trial. However, when comparing both groups with each other at each individual time point (baseline, just before travel and four days after travel), the WURSS-21 scores did not differ significantly at any time point, *i.e.*, the difference in profiles was not statistically significant (*p* = 0.18). The same analysis was conducted just for the participants with a defined cold, *n* = 29. Whilst, there was a tendency for increasing mean WURSS scores for elderberry and placebo participants across the three time points, the difference between both groups was not significant (*p* = 0.35).

**Figure 4:**
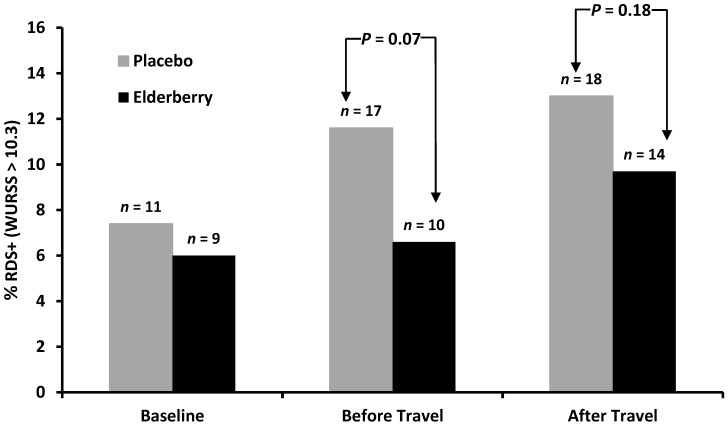
Effect of elderberry supplementation on the prevalence of Respiratory Disease Symptom positive (RDS+) participants.

When comparing the percentage of participants from the whole sample considering themselves to be affected by respiratory illness (RDS+, WURSS-21 score > 10.3) there was no significant difference between elderberry and placebo participants at any survey point (Figure 4). In both groups there is an increase of participants with significant symptoms (MID > 10.3). However, the survey completed immediately before travel and overseas showed a trend for more placebo participants reporting significant symptoms than elderberry participants, a trend that approaches significance at least for the before travel survey. When analysing only the cold sufferers who had a WURSS-21 score of >10.3 the numbers were too small to give reliable statistical data.

With regards to the secondary outcome measures, Quality of Life (QoL) measures, the individual scales of SF12 and their component summaries (physical and mental) were analyzed.

**Figure 5:**
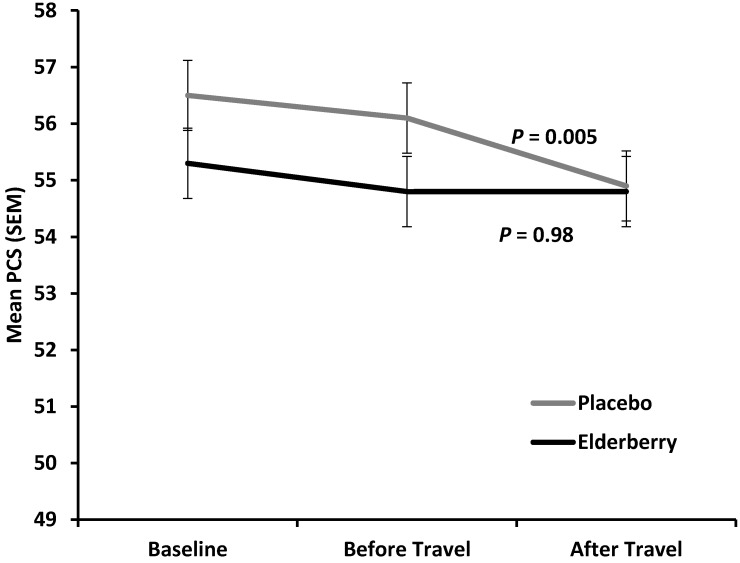
Y axis shows average Quality of Life (QoL) scores for physical health Physical Health Composite Scores (PCS) for each treatment group at the three time points surveyed (X-axis) for all participants.

Figure 5 shows the average change in physical health (reduction in mean Physical Component Score) for placebo and active group between baseline (−10 days) and overseas (+4/5 days).

The difference between placebo and active was minor and not statistically significant at overseas time point (*p* = 0.27), indicating that there is no treatment effect on change in physical health over the entire trial period. However, for the time of actual travel and arriving in a potential different climate and time zone (change observed from two days before until 4–5 days after travel) physical health declined significantly in the placebo group (*p* = 0.005), but was stable in the elderberry group (*p* = 0.9). Subdomain analysis for the PCS score (Physical Functioning (PF), Role Physical (RP), Body Pain (BP) and General Health (GH)) was performed to understand which subdomain(s) contribute(s) to this significant elderberry effect. There was a trend of PCS score rise for the three subdomains PF, RP and BP, but not GH. However, the change was not significant for any of the subdomains (data not shown).

**Figure 6:**
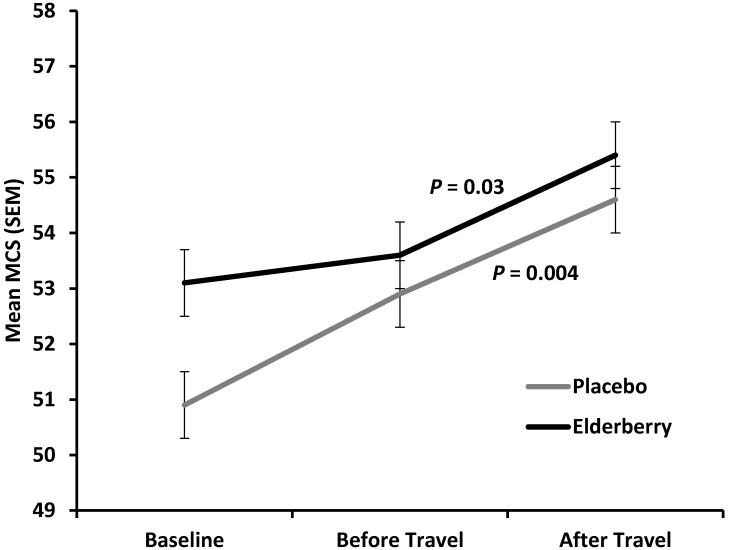
Y axis shows average QoL scores for mental health Mental Health Composite Scores (MCS) for each treatment group at the three time points surveyed (X-axis) for all participants.

Figure 6 shows an overall improvement of mental health (increase in mean Mental Component Summary score (MCS) for both, the placebo and active group from baseline (−10 days) to after travel (+4 days) with a non-significant trend for the active group to have a higher average mental health score compared to placebo (*p* = 0.07). Of note, the improvement in mental health was significant for both groups from travel to after travel (placebo, *p* = 0.004; elderberry group, *p* = 0.03). This indicates that mental health is strongly positively affected by travel whether people take elderberry or not.

Even though only 60% of participants were 100% compliant, *i.e.*, took all of their trial medication, more than 90% of participants were over 90% compliant. This means that 90% of trial participants did not miss more than three elderberry capsules throughout the whole trial period.

Moreover, no significant differences in participant’s compliance to the study medication were observed between the placebo and elderberry group (90% compliance: elderberry: *n* = 125; 92% *vs*. Placebo: *n* = 124; 92.5%, *p* = 0.6).

Overall, treatment was well tolerated. Five participants reported adverse events such as (i) itchy throat and cold-like symptoms (2 participants); (ii) fatigue (2 participants) and (iii) kidney pain (1 participant), however, a causal relationship between elderberry and the events could not be established. The two participants who reported itchy throat and cold-like symptoms were later unblinded as taking placebo and elderberry capsules, respectively. The two participants who reported fatigue were also later un-blinded as taking placebo and elderberry capsules, respectively. And, the participant who reported kidney pain was unblinded as taking placebo.

Individual sub-group analysis for participants that had no flu vaccination, travelled for more than 16 h, were more than 50 years old, fully complied with study medication schedule and were stressed, showed no significant differences between placebo and elderberry for any of the QoL indices for any of the survey time points (data not shown).

## 4. Discussion

In this study, participants in both, the placebo and elderberry group tended to experience an increase in respiratory symptoms from the time the trial started (baseline) to four days after arriving overseas. This was indicated by an increase in the prevalence of Respiratory Disease Symptom positive (RDS+) participants in both study groups. This result was expected as research has reported increased medical issues especially respiratory symptoms following commercial air travel independent of aircraft types [1].

Based on previous travel research we estimated the prevalence of cold episode days as 35% in the placebo group and hypothesized a ~50% prophylactic effect of elderberry (18%). Whilst we did observe a ~50% effect the prevalence of cold episode days in the overall study population was lower than expected (17% in the placebo and 8% in the active group). This is due to a lower than expected incidence of colds and reflects recent research that estimates the incidence of respiratory problems as a common complaint from air-travelers as ~11% [31], with in flight emergencies based on respiratory problems ranging from 2% to 14% [7]. Our trial is consistent with these recent studies identifying 29 participants (9%) as having a cold, defined by the Jackson score.

In our trial elderberry supplementation decreased the symptom load (mean 21 *vs*. 34) and shortened the cold duration by approximately two days (mean 4.75 days *vs*. 6.88 days). This supports the results from, previous limited research conducted with two other elderberry products, and shows for the first time that elderberry may be effective for decreasing respiratory symptoms during travel on long-haul flights. A reduction in the duration of symptoms has previously been reported for another elderberry formulation, Sambucol^®^, manufactured in Israel in two independent, but not travel related trials. Patients with either influenza A or B virus infection took 15 mL of elderberry syrup (38% extract equivalent to approx. 5.7 mg extract) four times a day for 5–6 days [16,32]. This formulation however, also contains honey and raspberry extract. A pilot trial with elderberry extract (175 mg) lozenges taken by patients with flu like symptoms four times for two days also confirmed a beneficial effect on severity and duration of cold and flu like symptoms [17].

Similarly, other complementary or nutritional medicines have shown to decrease cold duration. For example, a recent randomised clinical trial showed that supplementation with 1 g of vitamin C daily reduces cold duration by approximately three days [33]. Also, echinacea has been reported to reduce severity and cold duration [30,34], however more trials report a preventative effect on cold occurrences and re-occurrences rather than a conclusive treatment effect for echinacea [34,35]. In contrast, although our trial with elderberry also showed a lower incident of cold occurrences (*i.e.*, detected episodes) between the elderberry group and placebo, this difference was not significant. However, it may be that elderberry also has a weak preventative effect on the common cold which may have not been fully detected in our study due to sample size.

Similar to the Echinacea travel trial conducted by our group, the WURSS questions were used to detect impact on quality of life due to respiratory symptoms. In contrast to the Echinacea trial, the WURSS-21, which was not available then, was used in the elderberry study [11] to decrease survey completion times for participants as the WURSS-21 was reported to exhibit similar performance to WURSS-44 in terms of reliability, responsiveness, importance-to-patients, and convergence with other measures [25,27]. The WURSS-21 analysis of the cold sufferers (*n* = 29) did not show any significant differences between groups, probably due to sample size. However, it detected a weak elderberry effect in the overall sample on the prevalence of Respiratory Disease Symptom positive (RDS+) participants who are participants suffering from treatment worthy respiratory disorder symptoms (scores of 10.3 and above are RDS+). The difference between groups was not significant, but showed a trend especially at the “just before travel” and “overseas” time point. It would mean that travellers who take elderberry are less likely to suffer from respiratory symptoms that are worth treating.

Although there was a significant difference in symptom score (assessed with Jackson) and a slight difference in numbers of participants reporting treat worthy respiratory symptoms (assessed with WURSS-21) between both groups, this was not reflected by significantly increased co-medication of cold episodes or cold episode days in the placebo group. Nevertheless, most cold episodes in the elderberry group were co-medicated with one item, whereas most participants in the placebo group who suffered from a cold and co-medicated, used two and more medicines. More distinct was the result in a previously reported trial with Echinacea; a significant difference in co-medicated cold episodes was detected between the active and placebo group participants with a cold [30].

Intercontinental air travel can be stressful, adding extra strain on passenger’s physical health in general [1]. What effect elderberry has on physical health needs to be further investigated in larger studies, as so far the clinical evidence is very limited and diverse [36].

In our trial it seemed as if Elderberry “stabilizes” physical health (PCS score) from before travel until four days overseas, whereas the physical health in placebo participants significantly declined further. The effect was only noticeable when all PCS domains were considered in a holist (or additive) manner. When looking at individual PCS subdomains the effect was not detectable.

The slight effect of elderberry on physical health could be due to its reported antioxidant properties [37,38]. Antioxidant polyphenols are present in elderberries [22], bioavailable [39] and can increase serum antioxidant capacity [18]. In a recent randomised clinical trial it was shown that vitamin C supplementation improved physical activity levels in a population with adequate-to-low vitamin C status. The authors related this effect to vitamin C’s antioxidant properties since oxidative stress is related to fatigue [33].

Dosages recommended by popular elderberry products range from 650 mg to 1500 mg. In the three beneficial influenza trials patients took 60 mL of elderberry syrup (38% extract equivalent to approx. 22.8 mg extract standardised to flavonoids) daily for 5–6 days [16] or lozenges with an equivalent of 700 mg elderberry extract daily for two days with no indication on the anthocyanin content [17]. The minimum of anthocyanin doses for the treatment of metabolic syndrome disorders was recently estimated as 110 mg per day and as 3.5 g per day for influenza [40]. In our trial we used 600–900 mg of elderberry extract with 90–135 mg of anthocyanins daily and we recorded significant effects on severity and duration of respiratory symptoms and a possible stabilising effect on physical health.

While this study did not detect elderberry effects on mental health, it did show that going on holiday improves mental health. Over 80% of the participants in this trial travelled for leisure and this improved mental health (measured as MCS in SF-12) significantly, thus confirming psychological health effects of a vacation [41].

While raw and unripe fruit contain toxic cyanogenic glycosides, elderberries can be safely consumed when processed. Previous human studies and clinical trials using orally administered elderberry fruit extract reveal a very good safety profile [16,17,32]. Moreover, no adverse reaction reports were existent at the Advisory Committee on the Safety of Medicines (ACSOM, part of the Therapeutic Goods Administration (TGA) in Australia) prior to starting this trial [42].

In this trial, elderberry was generally well tolerated. For the adverse events reported by five participants no causal relationship between elderberry and the events could be established mainly due to the fact that participants unblinded to taking placebo. Due to only limited information for its use during pregnancy [43] we excluded any women who were pregnant or wanting to become pregnant. Moreover, we excluded travellers with plant allergies, due to one report on the possible allergenic potential of elder flowers and elderberry tree dietary products [44]. However, to our knowledge no allergic reactions to products using elderberry extract have been reported from any clinical trial, cohort study or government authority thus far. If this continues to be the case, further travel studies with elderberry products could include people with pre-existing plant allergies and respiratory conditions such as allergic rhinitis as this would mean that the trial population better represent the general traveller’s population and as such be more generalizable to a wider community.

As with all RCTs, this study had several limitations. Whilst diaries were used during the trial to help document cold symptoms and duration, as well as relief medication, a certain amount of recall bias has to be expected when the surveys were completed just before travel and after travel which contained the WURSS-21 and SF-12 questions. This study used travel including long haul intercontinental flights, time zone and climate change as a model to see whether elderberry is beneficial for respiratory and physical health overall. Generalisability of these results can only be extended to similar populations and season. In addition, this trial excluded travellers suffering from allergy and respiratory diseases (e.g., COPD, asthma, pneumonia) or immune disorders. As such effects and safety in these specific populations would need to be established in further studies.

## 5. Conclusions

Although the occurrence of the common cold for this trial in travellers was low overall, a significant effect of elderberry on cold duration and cold associated symptoms was detected. Travellers using elderberry from 10 days before travel until 4–5 days after arriving overseas on average experienced a 2-day shorter duration of the cold and also noticed a reduction in cold symptoms. Physical health may stabilise during air travel due to elderberry, but further studies are needed to confirm this. No elderberry effect was observed on mental health of the participants. The incidence of adverse events was low overall and no adverse effects could be directly attributed to elderberry. Based on these results it seems worthwhile to undertake more clinical research with high quality elderberry preparations to better understand beneficial health implications of this nutritional traditional medicine.

## Figures and Tables

**Table 1:** Demographics and characteristics of the overall sample.

Variable	Total (*n* = 312)	Elderberry (*n* = 158)	Placebo (*n* = 154)
Age in years—mean (SD) ^a^	51 (16)	52 (16)	50 (17)
Females (%)	206 (66)	108 (68)	98 (64)
Weight in kg (SD)	72 (14)	72 (15)	71 (14)
BMI (SD)	25 (4)	25(4)	25 (4)
Non-Smoker (%)	299 (96)	153 (98)	146 (95)
Perceived to be Stressed (%)	266 (85)	135 (86)	131 (85)
Received Flu Vaccination (%)	168 (54)	86 (54)	82 (53)
^b^ RDS+ (%)	20 (6)	9 (6)	11 (7)
Travel time > 16 h (%)	218 (70)	112 (71)	106 (69)
Travel Reason—Holiday (%)	255 (82)	131 (83)	124 (81)

^a^ Values are either means with standard deviations (SD) or frequencies with percentages (%); ^b^ RDS + indicates a Respiratory Disease Symptoms score of 10.3 and above.
